# Endpoint PCR coupled with capillary electrophoresis (celPCR) provides sensitive and quantitative measures of environmental DNA in singleplex and multiplex reactions

**DOI:** 10.1371/journal.pone.0254356

**Published:** 2021-07-23

**Authors:** Bettina Thalinger, Yannick Pütz, Michael Traugott

**Affiliations:** 1 Department of Zoology, University of Innsbruck, Innsbruck, Austria; 2 Centre for Biodiversity Genomics, University of Guelph, Guelph, Ontario, Canada; 3 Department of Integrative Biology, College of Biological Science, University of Guelph, Guelph, Ontario, Canada; 4 Sinsoma GmbH, Lannes 6, Völs, Austria; University of Hyogo, JAPAN

## Abstract

The use of sensitive methods is key for the detection of target taxa from trace amounts of environmental DNA (eDNA) in a sample. In this context, digital PCR (dPCR) enables direct quantification and is commonly perceived as more sensitive than endpoint PCR. However, endpoint PCR coupled with capillary electrophoresis (celPCR) potentially embodies a viable alternative as it quantitatively measures signal strength after PCR in Relative Fluorescence Units (RFU). Provided comparable levels of sensitivity are reached, celPCR permits the development of cost-efficient multiplex reactions, enabling the simultaneous detection of several target taxa. Here, we compared the sensitivity of singleplex and multiplex celPCR to dPCR for species-specific primer pairs amplifying mitochondrial DNA (COI) of fish species occurring in European freshwaters by analyzing dilution series of tissue extracts as well as field-collected water samples. Both singleplex and multiplex celPCR and dPCR displayed comparable sensitivity with reliable positive amplifications starting at two to 10 target DNA copies per μl extract. celPCR was suitable for quantifying target DNA and direct inference of copy numbers from RFU was possible after accounting for primer effects in linear mixed-effects models and calibration via dPCR. Furthermore, multiplex celPCR and dPCR were successfully used for the detection and quantification of fish-eDNA in field-collected water samples, confirming the results of the dilution series experiment and exemplifying the high sensitivity of the two approaches. The possibility of detection and quantification via multiplex celPCR is appealing for the cost-efficient screening of high sample numbers. The present results confirm the sensitivity of this approach thus enabling its application for future eDNA-based monitoring efforts.

## Introduction

DNA traces contained in environmental samples are frequently used for the detection of species in environmental studies and wildlife biology [1]. Recently, species detection from water samples using environmental DNA (eDNA)—DNA fragments released in the form of excretions, secretions, cell organelles, and free DNA into the environment [2] has transitioned from being a purely scientific method to being viewed as an alternative or complementary technique in routine species monitoring [3–7]. This creates a need for cost-efficient and reliable processing of large sample numbers.

Studies investigating the general species composition in environmental samples usually employ metabarcoding [6,8,9]. Individual species and their distribution are mainly investigated via targeted eDNA assays using endpoint PCR, quantitative real-time PCR (qPCR), or digital PCR (dPCR) [10–12]. For the amplification of eDNA, qPCR and dPCR are frequently complemented with probes to increase target-specific amplification. In addition, both techniques allow the quantification of target DNA [11,13]. Nevertheless, qPCR is an indirect approach as DNA quantities are calculated using standard curves and only dPCR enables direct and absolute quantification [14]. Endpoint PCR is also commonly used to detect target DNA from environmental samples. Although the visualisation of amplification success on agarose gels and the resulting binary (yes/no) data can be used for occupancy modelling [15,16], it does not generally allow quantitative estimates. This disadvantage can be compensated by analysing the endpoint PCR product via capillary electrophoresis (celPCR): in capillary electrophoresis, all double-stranded DNA fragments are separated by their size and each fragment is quantified in a relative manner by measuring its Relative Fluorescence Units (RFU). This is possible as either the primers or the whole fragment is fluorescently labelled [17,18]. In the past, celPCR has been used to determine if the fluorescence of a target amplicon exceeds a predefined threshold and samples can thus be scored “positive” [19,20]. Although the basic concept of this approach is not novel [21,22], there have been only rudimentary attempts to assess the general quantification capabilities of celPCR for eDNA analyses [18,23]. This possibility for quantification is especially appealing for target eDNA detection in large sample sets, as there is a high potential for cost-reduction based on PCR-chemicals alone (Table 1).

**Table 1:** Comparison of PCR reagent costs.

PCR-type	supplier	product name	reaction volume [μl]	Price (CAD)
celPCR	Qiagen	Multiplex PCR Kit	10	0.57
celPCR 5 targets multiplexed	Qiagen	Multiplex PCR Kit	10	0.11
dPCR	Bio-Rad	EvaGreen Supermix	20	1.38
		Supermix for Probes	20	1.38
qPCR	Thermo Fisher Scientific	TaqPath™ qPCR Master Mix, CG	20	1.57

Per reaction, costs between commonly used kits for dPCR, qPCR, and celPCR are compared (prices in CAD are calculated from lot sizes of 5,000 reactions combined with common reaction volumes; retrieved on 24^th^ October 2020).

Target DNA concentrations in environmental samples are usually low and therefore, the performance of both amplification and visualization methods at minute concentrations is crucial for successful detections [24]. To compare the sensitivity of assays, the Limit of Detection (LOD) is commonly used. However, its definition differs between PCR platforms: for qPCR, it is frequently defined as the target DNA concentration at which 95% of the reactions yield a positive result [25,26]. Theoretically, dPCR requires three out of 3,000 droplets to be positive, albeit the detection of single molecules is considered viable [27]. In practice, the LOD was found to be below 0.5 copies per μl in the dPCR mix [24,28]. For celPCR, the objective quantification of the fluorescence signal enables the definition of an LOD, which so far was defined as the amount of target DNA copies from which a reliable positive amplification (i.e. three or more positive replicates) is possible [17,29]. Endpoint PCR is sometimes associated with reduced sensitivity in comparison to qPCR and dPCR [30,31]. Yet, the LOD determined for invertebrate and vertebrate DNA with celPCR (10 to 30 target DNA copies in the reaction volume [17,18,29]) are similar to those found in qPCR (five to 50 copies in the reaction volume [30,32,33]). celPCR can therefore be considered sufficiently sensitive for detecting minute eDNA quantities.

Another aspect of targeted DNA amplification, which is hardly used in combination with eDNA detection, is multiplexing, i.e. the amplification of more than one target DNA fragment via the simultaneous use of several taxon-specific primer pairs [17,34]. Independent of the PCR platform and primer specificity, multiplex PCRs need to be balanced to exhibit similar levels of sensitivity for each of the contained primer pairs [17,35]. This can be achieved by designing primers with similar melting temperatures while minimizing cross-reactivity and competition among them [17,36]. It is also possible to adjust the concentration of specific primers or probes in PCR to counteract such effects [17]. In celPCR, multiplexing is accomplished by combining primer pairs yielding amplicons of different length [17,29,34]. However, such assays were so far not examined for any remaining effects of primer identity after the optimization process (e.g. via direct comparison with dPCR). Multiplex celPCR has been employed for the efficient screening of large sample sets to study trophic interactions [20,37], but not yet for eDNA studies. Albeit distinction via fragment length differences is also possible for qPCR and dPCR [38,39], this approach is rarely used. Instead, multiplexes on these instruments frequently employ specific dyes (attached to the respective probes) for each target, which does not require fragment length differences [36,39]. Due to the limited number of available dyes, their potential influence on primer/probe properties, and the rare use of length-based distinction in qPCR and dPCR [17,40], celPCR multiplexes present a straightforward alternative (but see [41] for a high-throughput qPCR approach). Generally, the use of multiplex PCR enhances the cost- and time-effectiveness of any screening for specific target taxa [17,29,36], but there has been no in-depth assessment whether this is possible for the detection of eDNA without forfeiting sensitivity.

We designed species-specific primers for the mitochondrial cytochrome *c* oxidase subunit I (COI) gene of seven freshwater fish species occurring in Central Europe and optimized amplification conditions for singleplex celPCR, dPCR, and two multiplex celPCR. The sensitivity was compared between the three approaches via a dilution series experiment, which also evaluated the potential to quantify target eDNA from celPCR results. Finally, field-collected water samples were analyzed with multiplex celPCR and dPCR to evaluate eDNA quantification with celPCR and to compare the sensitivity of the two approaches. We investigate the possibility to estimate target DNA copy number from RFU obtained by celPCR and hypothesize that primer identity affects PCR efficiency even if primer characteristics are chosen for maximum similarity between primer pairs. Finally, we test whether multiplex celPCR is sufficiently sensitivity to detect and quantify eDNA of target species in field-collected samples.

## Materials and methods

All laboratory work was carried out in a clean-room laboratory at the University of Innsbruck, which was equipped with an ultraclean overpressure air system, separate rooms for DNA extraction, PCR preparation, thermo-cycling and post-PCR work, always using laminar flow workbenches, DNA-free gloves and protective clothing. All surfaces were cleaned with 10% bleach and 70% ethanol prior to laboratory work and all workbenches were daily radiated with UVC-light for three hours.

### Primer design and PCR optimization

Species-specific primers were designed for seven fish species commonly occurring in rhithral freshwaters in Central Europe, namely *Cottus gobio*, *Oncorhynchus mykiss*, *Salvelinus fontinalis*, *Salvelinus umbla*, *Salmo trutta*, *Squalius cephalus*, and *Thymallus thymallus*. For this task, a custom reference sequence database containing the COI sequences of all Central European freshwater fish species was used [29]. Suitable priming regions were identified using BioEdit Version 7.3.5 [42] before using Primer Premier 5 (PREMIER Biosoft International) to design species-specific primer pairs with melting temperatures as close as possible to 60°C, amplicon lengths between 89 and 226 bp, and minimizing potential formation of dimers and secondary structures. After initial singleplex PCR testing, primer pairs were arranged in two multiplex PCR assays with at least 20 bp length difference between amplicons, enabling target identification based on amplicon length in capillary electrophoresis. Multiplex PCR conditions were optimized and primer concentrations adjusted to obtain similar sensitivity and amplification efficiency across all primer pairs using standardized DNA templates [17,18,29]. The final singleplex and multiplex PCRs underwent specificity testing using muscle tissue extracts from Central European fish species focusing on the seven target fish species, closely related species, and species with only a small number of mismatches at the respective priming sites. In total, we tested each primer pair with 21 to 29 non-target species. Two to three extracts were used per species (see S1 File for an alignment of target species, non-target species, and primers). Primers were found to be species-specific as no non-target amplification occurred with the below reported PCR conditions and non-target extract concentrations of 10 to 165 ng/μl (mean 38.3 ng/μl ± 35.3 ng/μl SD; measured via NanoDrop [Thermo Fisher Scientific]).

Both singleplex and multiplex endpoint PCR assays were based on the Multiplex PCR Kit (Qiagen) and contained bovine serum albumin (BSA) and tetramethylammonium chloride (TMAC) to reduce inhibition and enhance specificity [43,44]. Each 10 μl reaction contained 1 × reaction mix, 5 μg BSA, 30 mM TMAC, the respective primer combinations (Table 2) and 3.2 μl extract. For the dilution series experiment, the master mix was altered by using only 1 μl extract (or its respective dilution) and adding 2.2 μl molecular grade water. The thermocycling conditions with optimum sensitivity and specificity on a Mastercycler® nexus (Eppendorf) were 15 min at 95°C, 35 cycles of 94°C for 30 s, 65°C for 3 min and 72°C for 60 s and final elongation at 72°C for 10 min. For amplicon separation and visualization after endpoint PCR, the capillary electrophoresis system QIAxcel Advanced and the software QIAxcel ScreenGel (version 1.4.0, Qiagen) with the method AM320 and 30 s injection time were used. If PCR products of the expected fragment length reached a signal strength ≥ 0.08 RFU, they were deemed positive and their RFU were recorded. This detection threshold was employed for both singleplex and multiplex celPCR. It enabled the clear distinction of successful amplifications from background fluorescence and was chosen based on previously used thresholds (0.07 and 0.1 RFU [29,45]) and after reviewing background signals in PCR controls and extraction negative controls. The singleplex and multiplex celPCRs were run in 96-well plates and contained at least two negative and two positive controls (approx. 100 DNA copies per target species and reaction). All negative controls resulted negative; all positive controls delivered the expected target amplicon(s). Albeit the *Salvelinus umbla* primer pair was included in one of the optimized multiplex reactions, it was not used in any of the consecutive processes (i.e. optimization on the dPCR platform, dilution series experiment) and the species was never detected in field-collected samples.

**Table 2:** The target fish species and the associated species-specific primer pairs.

Species	Primer name	5’ - 3’	Target gene	fragment length (bp)	MP #	Concentration in MP (μM)	Concentration in SP (μM)
*Salmo trutta*	Sal-tru-S1002	TCTCTTGATTCGGGCAGAACTC	COI	89	1	0.4	0.5
	Sal-tru-A1002	CGAAGGCATGGGCTGTAACA			1	0.4	0.5
*Salvelinus fontinalis*	Salfon-S715	CCTCCCGCCCTCCTTTCTA	COI	152	1	0.45	0.5
	Salfon-A715	TGCCAGCTAAATGTAGGGAAAAA			1	0.45	0.5
*Thymallus thymallus*	Thythy-S720	GGAGCCCTTCTGGGTGATGAT	COI	226	1	0.2	0.5
	Thythy-A720	TTCAACCCCAGATGAGGCTAAG			1	0.2	0.5
*Oncorhynchus mykiss*	Oncmyk-S714	ATAAAACCTCCAGCCATCTCTCAG	COI	94	2	0.4	0.5
	Oncmyk-A714	GGACGGGGAGGGAAAGTAAYAG			2	0.4	0.5
*Salvelinus umbla*	Salumb-S717	GCTTCTGACTCCTCCCACCG	COI	142	2	0.15	0.5
	Salumb-A717	AAGATAGTTAAATCAACGGAGGCC			2	0.15	0.5
*Squalius cephalus*	Squcep-S719	TCGGAAACTGACTTGTCCCG	COI	184	2	0.15	0.5
	Squcep-A719	GCGTGAGCAAGATTGCCC			2	0.15	0.5
*Cottus gobio*	Cotgob1-S712	GAAGCAGGTGCCGGAACC	COI	206	2	0.4	0.5
	Cotgob1-A712	GATCATACGAAGAGCGGGGTC			2	0.4	0.5

The target gene, fragment length, association with one of the two multiplex assays (MP) and the respective primer concentrations in multiplex and singleplex celPCR are provided.

In a next step, the primer pairs (Table 2; exception: *S*. *umbla*) were used to create EvaGreen-based droplet dPCR assays using the AutoDG (Bio-Rad) for droplet generation, a Mastercycler® nexus for amplification, and the QX200 Droplet Reader with its corresponding software QuantaSoft 1.0.596. (Bio-Rad) for fluorescence detection. We optimized dPCR conditions by adjusting annealing temperature and/or time, and by using three-step protocols with separated annealing and extension phases to obtain a clear separation of positive and negative droplets and minimum “rain” (i.e. droplets with intermittent fluorescence between positive and negative droplets). Subsequently, a non-target test was conducted using the respectively other species and the three Central European fish species with the least sequence divergence at the priming sites. Ultimately, each 22 μl reaction mix, of which approx. 20 μl were used in the droplet generation process, contained 1 × EvaGreen Supermix (Bio-Rad) and 113.6 nM of each primer (Table 2). The remaining 10.5 μl reaction volume were filled with 8.3 μl molecular grade water and 2.2 μl extract in the dilution series experiment, and varying extract volumes for the testing of field-collected samples. The optimum dPCR thermocycling conditions were 95°C for 15 min, 40 cycles of 95°C for 30 s, 58°C (*O*. *mykiss* and *S*. *fontinalis*), or 60°C (*S*. *trutta* and *S*. *cephalus*), or 62°C (*T*. *thymallus*), or 64°C (*C*. *gobio*) for 60 s, and 72°C for 60 s, followed by stabilization at 4°C for 5 min, 90°C for 5 min, and 12°C until further processing on the droplet reader. It was necessary to manually set a threshold for positive droplets for each target species, as the fluorescence levels varied with the fragment length generated by the respective primer pair. This threshold separates positive from negative droplets [10,46] by specifying the lowest fluorescence signal that is distinct from background noise [24]. We chose to set a conservative threshold right below the cloud of positive droplets [46]. For *C*. *gobio* the threshold was set at 20,200 amplitude, for *O*. *mykiss* at 13,100, for *S*. *fontinalis* at 15,000, for *S*. *trutta* at 16,100, for *S*. *cephalus* at 18,300 and for *T*. *thymallus* at 18,400. All samples were processed in 96-well plates along with at least two positive and two negative controls, all of which resulted positive or negative, as expected.

### Dilution series experiment

The template DNA concentration of one extract each of *C*. *gobio*, *O*. *mykiss*, *S*. *fontinalis*, *S*. *trutta*, *S*. *cephalus*, and *T*. *thymallus* was measured three times at the respective dPCR conditions. Based on these results, the extracts were diluted to 5,000 target DNA copies per μl using 1 × TE buffer. Then, a dilution series with 21 steps (5,000; 4,000; 3,000; 2,000; 1,500; 1,000; 750; 500; 400; 300; 200; 150; 100; 80; 60; 40; 30; 20; 10; 5; 1 copy per μl) was generated for each species. Each of the dilutions was used nine times: for three replicates of singleplex celPCR, multiplex celPCR, and dPCR each. The PCRs and the visualization of the obtained results were carried out right after setting up the dilution series. Cooling racks were used during dilution and PCR preparation; diluted extracts were not frozen during processing. Throughout the experiment, each dPCR reaction produced more than 15,600 droplets (total) and the resulting DNA concentrations were converted into target copies per μl in the original or diluted DNA extract (henceforth “copies per μl extract”).

### Field samples

Field-collected samples were obtained as part of a larger study (in prep.) during which electrofishing and water sampling for eDNA detection were carried out simultaneously at 16 rivers of varying size (70 L/s to 2,310 L/s discharge) in Tyrol (Austria). For each sample, 2 L of water were collected in DNA-free wide-neck bottles and filtered in the field through 47 mm glass fiber filters with 1.2 μm mesh width (Whatman GF/C) using a peristaltic pump (Solinst, Model 410). Filters were transported in cooling boxes to the University of Innsbruck and stored at –20°C until further processing. Lysis and DNA extraction were carried out as described by Thalinger et al. [18]: the filters were incubated overnight in lysis buffer before separating the lysate from the filter by centrifugation and extracting the DNA using the Biosprint 96 robotic platform (Qiagen).

All field samples were analyzed using the two multiplex celPCR assays (Table 2) and capillary electrophoresis. For the herewith presented analysis, we randomly selected for each target species 25 samples testing positive and five samples testing negative in multiplex celPCR (out of 334 samples analyzed as part of the large field study; in prep.). These were analyzed with dPCR using the optimized conditions described above. To avoid background fluorescence from non-target DNA contained in the field sample extracts, 2.63 μl of extract was used per dPCR reaction for samples with RFU above 0.5, 5.25 μl were used for samples with RFU between 0.21 and 0.5, and 10.5 μl of extract was analyzed in case of RFU between 0.08 and 0.2 to ensure a positive amplification despite very low target DNA concentration. As background fluorescence varied between samples from different locations, it was necessary to manually adjust the fluorescence threshold for positive droplets, albeit the positive and negative droplet clouds were clearly distinguishable for all samples.

### Statistical analysis

All calculations and visualizations were made in R Version 4.0.2 [47] using the packages “ggplot2” [48], “ggpubr” [49], “outliers” [50], “lme4” [51], “nlme” [52], “MuMIn” [53], “caret” [54] and “ie2misc” [55].

First, the RFU from the singleplex and multiplex celPCR and the copy numbers calculated with dPCR were plotted against the expected copy numbers of the dilution series. Limits of Detection (LODs, i.e. the lowest number of target copies for which positive amplifications occurred; inferred from triplicate dPCR measurement) and Limits of Quantification (LOQs, i.e. all three replicates lead to a positive amplification) were evaluated for singleplex and multiplex celPCRs following Agersnap et al. [56] as it was not possible to directly transfer the LOD definition recently established by Klymus et al. [25] to this experiment. Prior to any other analyses, Grubbs’ tests were performed to remove outliers from the triplicate measurements [57]. Additionally, the lowest dilution was removed from the dataset, as not all replicates tested positive on all PCR platforms. For each dilution step, the means and standard deviations of RFU (measured using single- and multi-plexed celPCR) and copies per μl extract (measured using dPCR) were calculated. Based on the mean RFU, PCR efficiency was compared between singleplex and multiplex celPCR using linear models. Then, the relationship between RFU and copies per μl extract was evaluated using linear mixed-effects models (LMM). The natural logarithm of mean copies per μl extract was entered as dependent variable, while mean RFU derived from either singleplex or multiplex celPCR were entered as fixed effect, and fish species as random effect (random slope and intercept). As a next step, we examined the fluctuation of the model fit and the error rate of such LMM when applied to previously unconsidered tissue extract dilutions using a validation set approach. The dilution series dataset was 200 times randomly split in a 70:30 ratio. Per training dataset (70%), an LMM describing the relationship between RFU and copies per μl extract was generated separately for singleplex and multiplex celPCR results. These models were used to predict copy numbers in the respective test datasets (30%), compare the predicted and measured values, and determine model accuracy via the Root Mean Square Error (RMSE). For the field-collected samples, we first predicted copy numbers from both the LMM based on the entire dilution series dataset (LMM_full_) and the LMM with the highest predictive power derived from the 200 subsets (LMM_max70_, both based on multiplex celPCR), and compared their accuracy. Finally, linear models describing the relationship between *ln*-transformed copies and RFU in field samples were calculated and observed and predicted copy numbers were plotted together with data obtained from the dilution series experiment.

## Results

**Fig 1:**
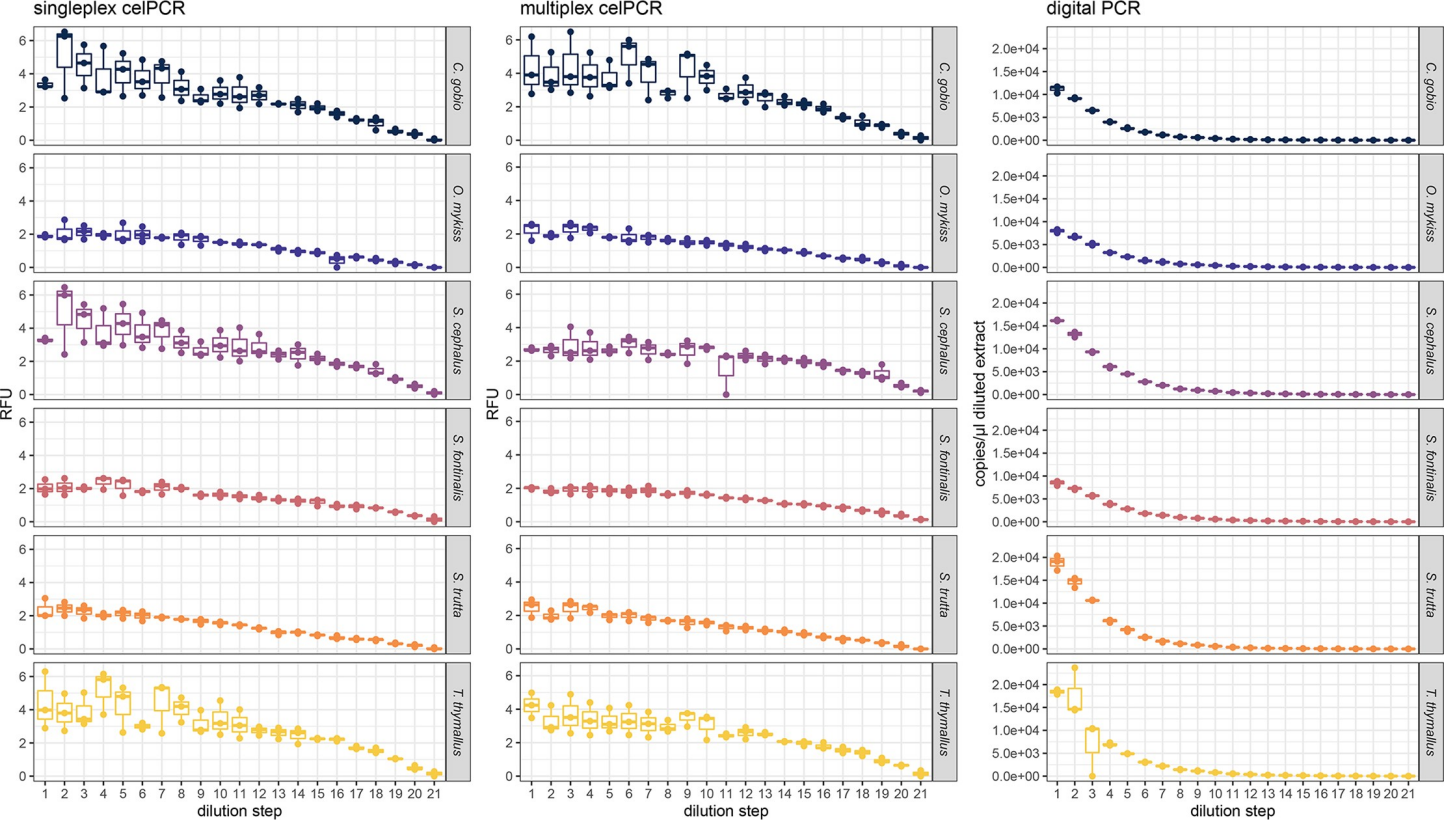
Singleplex and multiplex celPCR and dPCR dilution series. Relative Fluorescence Units (RFU) and template DNA copy numbers per μl diluted extract obtained for *C*. *gobio*, *O*. *mykiss*, *S*. *fontinalis*, *S*. *trutta*, *S*. *cephalus*, and *T*. *thymallus* from singleplex celPCR, multiplex celPCR, and dPCR. Dilution steps from 5,000 copies to 1 copy per μl extract are abbreviated 1 to 21.

In the dilution series experiment, the target DNA concentration per μl diluted extract was quantified via dPCR for each of the six target species from a maximum of 23,680 copies to a minimum of 0.6 copies. Diluted extracts tested positive for all species with both singleplex and multiplex celPCR, with RFU ranging from 0.09 to 6.53 in singleplex celPCR and 0.09 to 6.48 in multiplex celPCR, respectively. RFU showed an exponential decline with increasing dilution, and generally higher levels of variability (especially at higher DNA concentrations) compared to dPCR (Fig 1).

**Fig 2:**
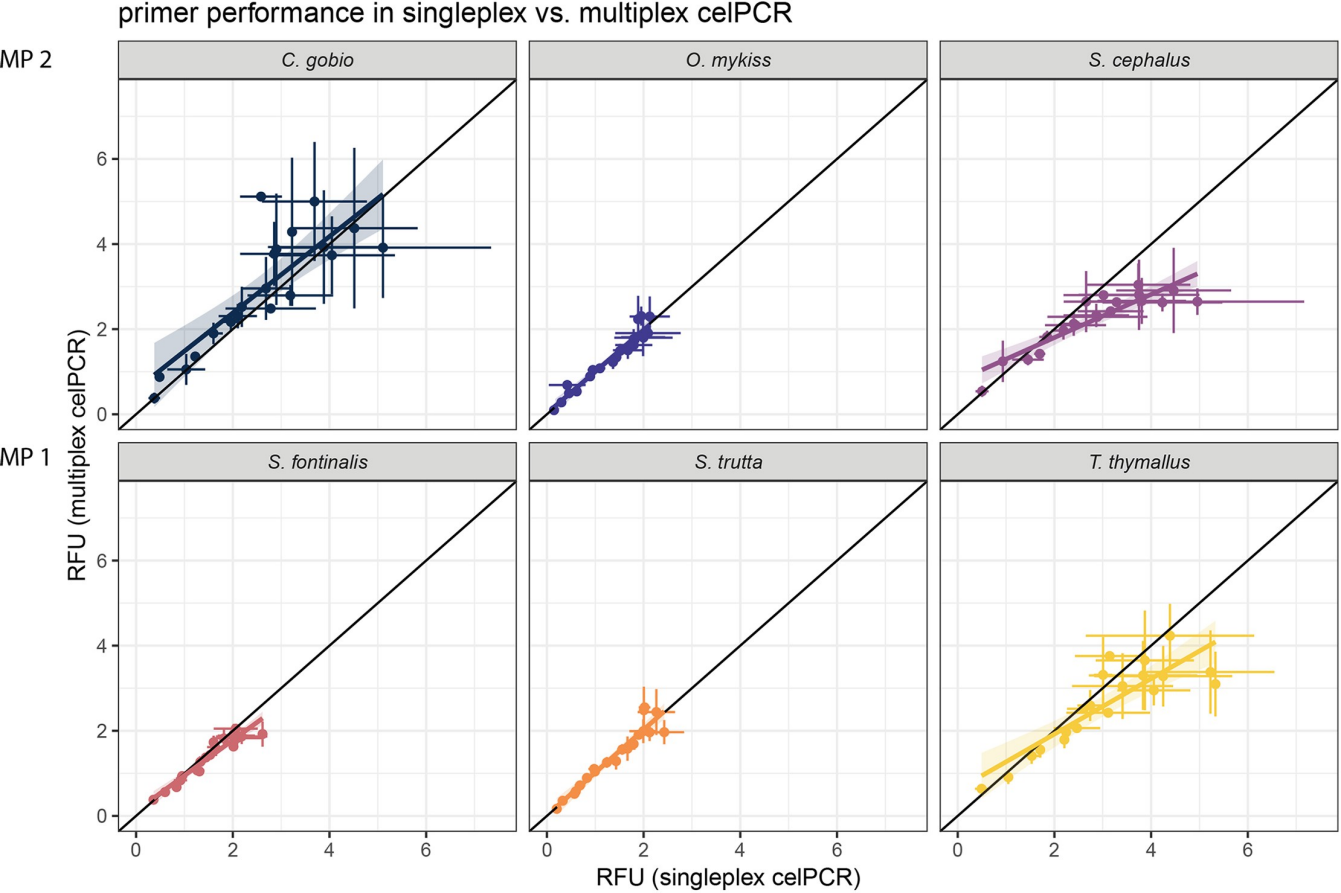
Singleplex vs. multiplex celPCR amplification. Visualizations and calculations are based on mean RFU (dots). The corresponding standard deviations are displayed as whiskers; the shaded area depicts the 95%-CIs; see S2 File for model specifications.

**Fig 3:**
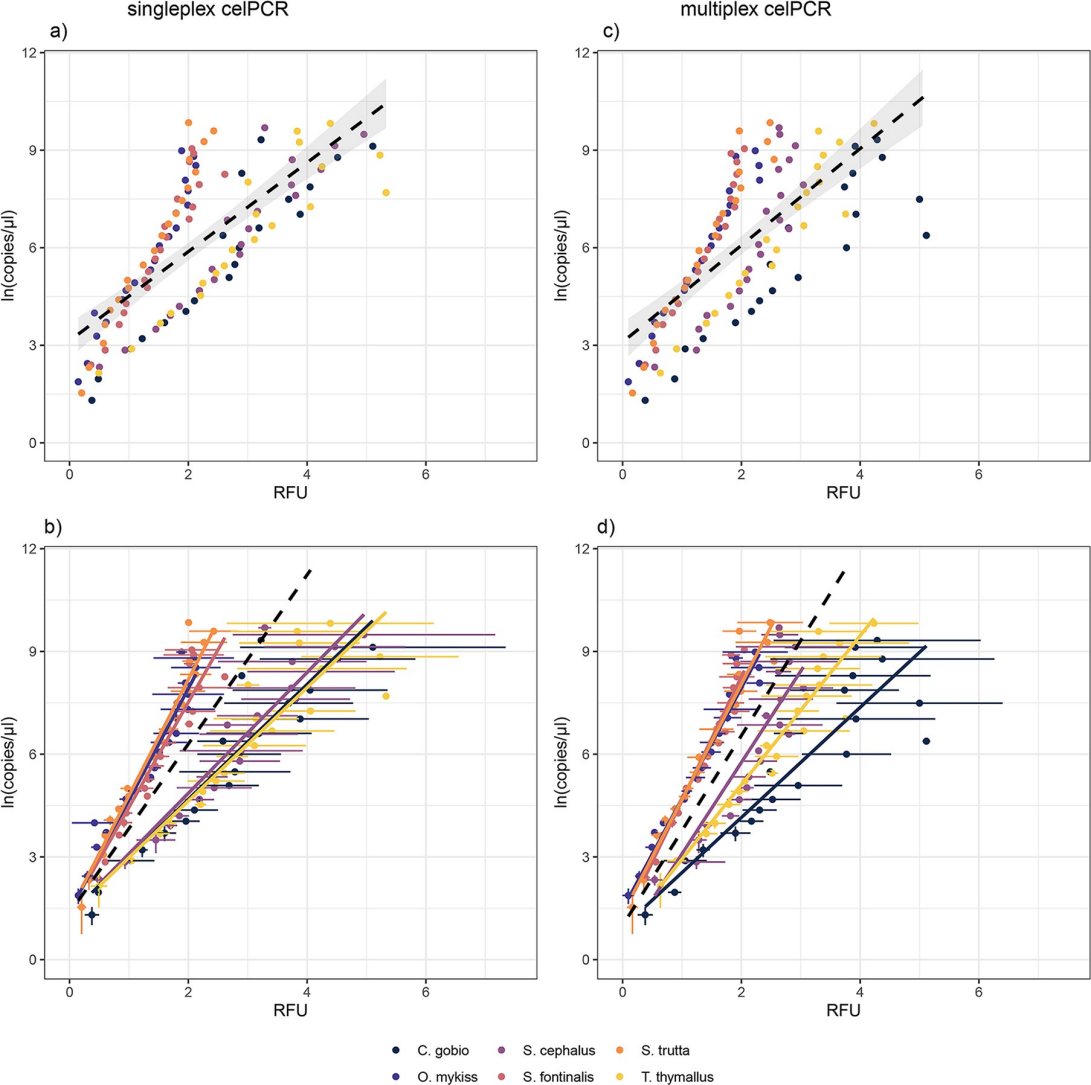
Linear models and linear mixed-effects models (LMM) for singleplex and multiplex celPCR in relation to *ln*-transformed copy numbers per μl extract. Panels a) and c) display mean RFU and copy numbers per dilution step. Values are color coded by species, the black dashed line represents a linear model fitted onto this dataset without accounting for target species identity, the shaded area depicts the 95%-CIs; see S3 File for model specifications. Panels b) and d) show the LMM using target species identity as random effect and permitting random slope and intercept (Table 3). Dots represent mean RFU and copy numbers per dilution step, with the corresponding standard deviations displayed as whiskers. The black dashed line depicts the linear model of the population mean; colored lines are the slopes associated with the individual species.

Amplification efficiency differed significantly between singleplex and multiplex celPCR for *S*. *cephalus*, *S*. *fontinalis*, and *T*. *thymallus*, with multiplex reactions leading to higher signal strengths at low DNA concentrations and singleplex reactions resulting in elevated RFU at high DNA concentrations (Fig 2, S2 File). This trend was not observed for the three other species. The R^2^ of the linear regressions describing the relationship between RFU obtained from singleplex and multiplex celPCR ranged from 0.68 (*C*. *gobio*) to 0.93 (*O*. *mykiss*; S2 File). The comparison of RFU (singleplex or multiplex celPCR) to copy numbers per μl diluted extract (dPCR) showed amplification differences between primer pairs in endpoint PCR (Fig 3A and 3C). After accounting for primer pair identity, *ln*-transformed copy numbers per μl extract could be estimated from singleplex and multiplex RFU (conditional R^2^ = 0.96 for both LMM; Table 3, Fig 3B and 3D). In both the singleplex and the multiplex celPCRs, the RFU produced by *C*. *gobio*, *T*. *thymallus*, and *S*. *cephalus* primers were above the population mean (Fig 3).

**Table 3:** Linear mixed-effects models for singleplex and multiplex celPCR.

**Singleplex PCR (Model 1)**	**Random effects**			**Variance**		**Standard deviation**
						
	*intercept*			0.002		0.045
	Mean SP PCR RFU			0.73		0.85
						
	**Fixed effects**	**parameter estimate**	**lower 95% CI**	**upper 95% CI**	**t-value**	**p-value**
						
	*intercept*	1.36	1.03	1.69	8.09	< 0.001***
	Mean SP PCR RFU	2.47	1.76	3.18	6.89	< 0.001***
						
	**Estimated deviation**	**species**	**intercept**	**Mean SP PCR RFU**		
		*C*.* gobio*	–0.04	–0.79		
		*O*.* mykiss*	0.04	0.79		
		*S*.* fontinalis*	0.03	0.60		
		*S*.* trutta*	0.04	0.92		
		*S*.* cephalus*	–0.03	–0.70		
		*T*.* thymallus*	–0.04	–0.81		
**Multiplex PCR (Model 2/LMM**_**full**_**)**	**Random effects**			**Variance**		**Standard deviation**
						
	*intercept*			0.24		0.49
	Mean MP PCR RFU			0.63		0.79
						
	**Fixed effects**	**parameter estimate**	**lower 95% CI**	**upper 95% CI**	**t-value**	**p-value**
						
	*intercept*	0.99	0.46	1.53	3.66	< 0.001***
	Mean MP PCR RFU	2.77	2.11	3.44	8.24	< 0.001***
						
	**Estimated deviation**	**species**	**intercept**	**Mean MP PCR RFU**		
		*C*.* gobio*	–0.05	–1.17		
		*O*.* mykiss*	0.47	0.46		
		*S*.* fontinalis*	0.06	0.83		
		*S*.* trutta*	0.33	0.58		
		*S*.* cephalus*	–0.57	–0.11		
		*T*.* thymallus*	–0.24	–0.60		

**Fig 4:**
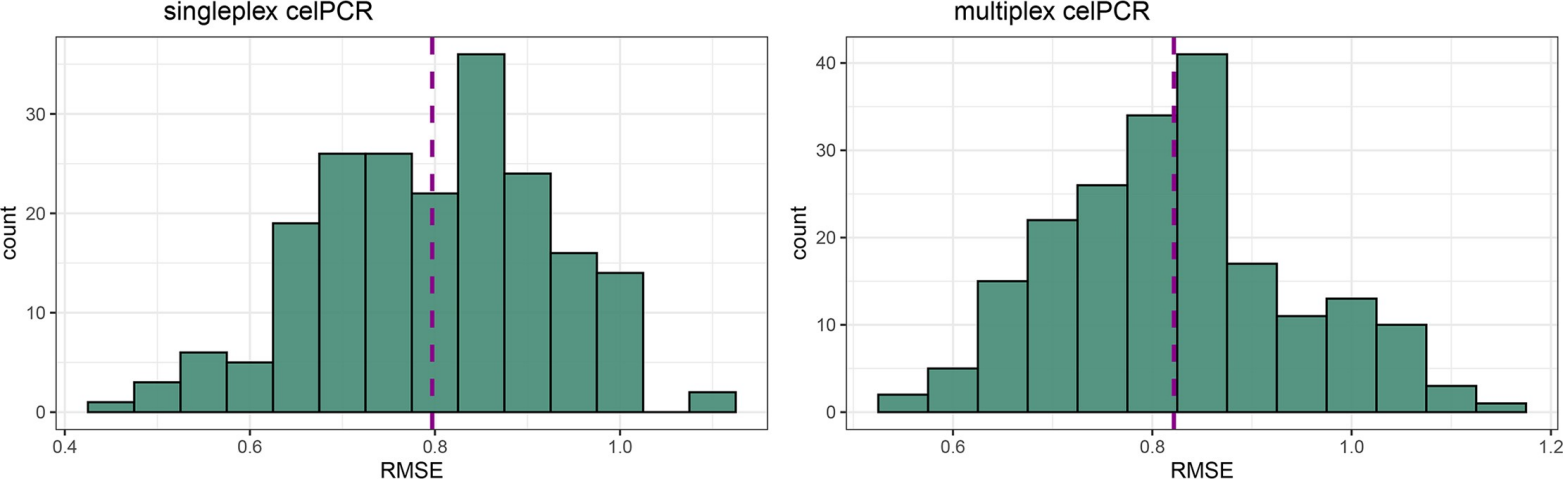
Distribution of Root Mean Square Error (RMSE) for predictions based on singleplex and multiplex celPCR. Histograms of RMSE are derived from 200 random 70:30 splits of the dilution series dataset. Linear mixed-effects models were established for each subset using the 70% share and tested on the respective 30% subset for singleplex celPCR (left column) and multiplex celPCR (right column). Dashed lines indicate mean RMSE (singleplex: 0.80; multiplex: 0.82).

The evaluation of LMMs generated from 200 70%-subsets of the dilution series data and tested on the remaining 30%-share of the dataset generated RMSE from 0.47 to 1.1, and from 0.55 to 1.16 for predictions based on singleplex celPCR and multiplex celPCR, respectively (Fig 4). The RMSE showed a slightly lower variation for the singleplex celPCR, (singleplex mean 0.80; multiplex mean 0.82).

The lowest DNA concentrations which produced positive amplifications (≥ 0.08 RFU; LOD) in singleplex and multiplex celPCR contained target DNA quantities as measured via dPCR from 0.6 to 8.1 copies per μl diluted extract. The LOQs in both singleplex and multiplex celPCR inferred from triplicate dPCR measurements covered concentrations from 0.6 to 13 copies per μl diluted extract (Table 4). As singleplex and multiplex PCRs both contained 1 μl of diluted extract, copies per μl are equivalent to copies in PCR.

**Table 4:** Species-specific LOD and LOQ in singleplex and multiplex celPCR.

species	LOD [copies/μl]	LOQ [copies/μl]
*Cottus gobio*	0.7	3.1–4.8
*Oncorhynchus mykiss*	6.5–8.1	5.1–8.0 (singleplex)
		9.4–13 (multiplex)
*Squalius cephalus*	0.6–2.4	9.1–12 (singleplex)
		0.6–2.4 (multiplex)
*Salvelinus fontinalis*	0.6–1.3	5.6–11 (singleplex)
		0.6–1.3 (multiplex)
*Salmo trutta*	0.6 (singleplex)	2.3–7.3
	2.3–7.3 (multiplex)	
*Thymallus thymallus*	1.8–2.4	5.2–13

The LOD (lowest target DNA amount with amplification) and LOQ (lowest target DNA amount with all technical replicates yielding a positive result) of multiplex and singleplex celPCR are displayed for the individual target species.

Of the five field samples per target species which tested negative in multiplex celPCR, all but two were also negative in dPCR (one positive for *S*. *trutta* with 0.25 copies per μl extract and one positive for *C*. *gobio* with 0.13 copies per μl extract). The LMM_full_ and LMM_max70_ (see S4 File for model summary) had a similar accuracy for predicting target DNA copies in field-collected water samples from RFU. The RMSE of LMM_full_ was 8% lower than for LMM_max70_ (1.06 versus 1.13).

**Fig 5:**
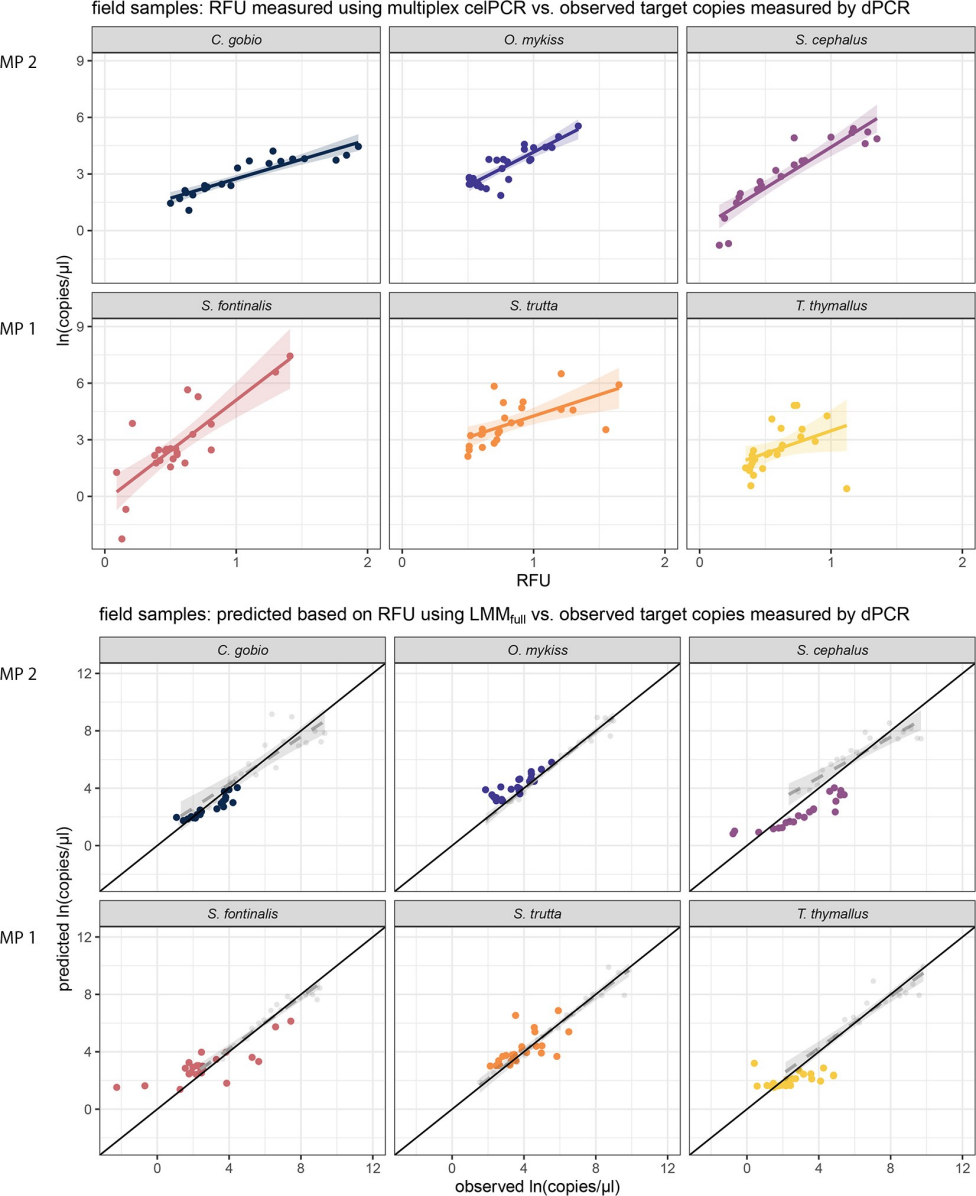
Multiplex celPCR and dPCR results and predicted vs. observed DNA concentrations for field-collected samples. The relationship between RFU and *ln*-transformed target DNA copy numbers measured in field-collected samples for each of the primer pairs is displayed in the upper panel; for details on the linear models and their 95%-CIs see S5 File. The relationship between target DNA copy numbers measured in field-collected samples in comparison to the predicted copy numbers based on LMM_full_ is depicted in the lower panel. The black line (origin 0/0, slope 1) represents a perfect fit between observed and predicted copy numbers; the dashed regression line and the associated 95%-CIs are based on a comparison between measured and predicted copy numbers from the dilution series experiment based on LMM_full_ and mean RFU per dilution step. For details on the linear models and 95%-CIs see S6 File.

The linear models describing for each primer pair the relationship between RFU and *ln*-transformed copy number in field-collected samples showed different R^2^ levels ranging from 0.13 to 0.82 (S5 File, Fig 5 upper panel). When comparing the relationship between observed and predicted copy numbers, data based on field-collected samples and the dilution series overlapped for *C*. *gobio*, *O*. *mykiss* and *S*. *trutta* (Fig 5 lower panel). However, for all six primer pairs, the dispersion was higher for data derived from field-collected samples than for the dilution series data. Ultimately, the observed and predicted copy numbers obtained from field-collected samples represent only a small part of the range examined via the dilution series and for *C*. *gobio*, *S*. *cephalus*, *S*. *fontinalis*, and *T*. *thymallus* align themselves at or beneath the lowest concentrations used in the experiment (Fig 5 lower panel).

## Discussion

Our results demonstrate the capacity of celPCR to provide a quantitative analysis of target eDNA copy numbers. After considering primer identity for singleplex and multiplex celPCR and modeling the relationship between the RFU and copies per μl extract (measured using dPCR) with LMM, it was possible to predict target DNA concentrations in diluted extracts with a mean RMSE of 0.80 (singleplex) versus 0.82 (multiplex) in randomly generated subsets. Furthermore, both singleplex and multiplex celPCR displayed high levels of sensitivity for detections from diluted tissue extracts and field-collected eDNA samples, thus enabling the future application of cost-efficient multiplexes in large-scale screenings.

The comparison of DNA concentrations measured directly via dPCR to the RFU measured via celPCR displays the exponential nature of endpoint PCR [19,58]. The diluted extracts processed simultaneously and in triplicate with both approaches showed increasing signal strength variability with increasing target DNA concentration. This is due to the endpoint reaction not being split into thousands of separate reactions [10]; hence, slight differences in DNA quantities at the start of the reaction can have strong effects on the final signal strengths. The signal strength in celPCR is also subject to saturation effects commonly occurring in the later stages of PCR and caused by template re-annealing, exhaustion of dNTPs or primers, or loss of polymerase activity [59]. In our experiment, these two effects were primarily visible for RFU > 3. In the field samples under investigation, RFU were never > 2 and higher signal strengths seem to be hard to reach in field-collected samples from rivers in temperate climate regions [18,23]. Nevertheless, other factors such as inhibition can potentially affect measurements derived with celPCR and (less likely) dPCR [60] from field-collected samples. We observed higher levels of dispersion and varying R^2^ for individual species when comparing the results of dPCR and multiplex celPCR for field-collected samples. Thus, we recommend investigating inhibition effects prior to any large-scale field sampling campaign relying on multiplex celPCR. Additionally, PCRs of field-collected samples should be carried out in triplicate for accurate quantification, especially if higher target DNA concentrations are expected.

For the prediction of absolute target DNA concentrations from RFU it was necessary to account for primer effects, albeit the primer pairs were designed for equal amplification efficiency at uniform PCR conditions. As previously recommended [17,36], melting temperatures were as close as possible to 60°C and the variation in fragment length (89–226 bp) was kept as small as possible and within the general suggestion for the detection of low concentrations of potentially degraded DNA from mixed samples [34]. The selected primers displayed minimal secondary structures and no competition for priming sites [17,36], and the multiplex PCRs were calibrated for equal amplification efficiency by adjusting primer concentrations in tests with target DNA templates [17,29]. However, all these measures were not sufficient to completely eliminate primer bias *a priori* for both singleplex and multiplex PCR. A direct estimate of target DNA concentration was made possible by relating the RFU to absolute concentrations measured via dPCR and accounting for primer effects in LMM. In our dilution series experiment, LMM generated from 200 randomly drawn subsets containing 70% of the dilution series data could predict copy numbers in the respective test datasets (30%) for both singleplex and multiplex celPCR with a mean RMSE of 0.80 (singleplex) and 0.82 (multiplex), respectively. This indicates accurate prediction of copy numbers from RFU. However, the exponential nature of PCR and the concomitant *ln*-transformed of copy numbers in our models imply that for high RFU even a small difference between predicted and observed *ln*-transformed copy numbers leads to inaccurate predictions. In practice, the low eDNA concentrations in field-collected samples (RFU < 2) will limit this effect and thus, absolute DNA concentrations can be deduced from RFU if the efficiency of the applied primer pair(s) is directly compared between celPCR and a PCR type enabling absolute quantification (i.e. dPCR). Despite careful design, the amplification efficiency of a specific primer pair can differ between singleplex and multiplex PCR and individual primer concentrations need to be adjusted such that the amplification efficiency is similar for each primer pair in balanced multiplex reactions [17]. Thus, screenings incorporating quantitative estimations from celPCR require a thorough evaluation of each specific celPCR assay using qPCR or dPCR.

Both singleplex and multiplex celPCR displayed similar levels of sensitivity in our experiments and resulted in positive amplifications of all reaction triplicates at concentrations between two and 13 target copies per μl diluted extract (equalling two to 13 copies per 10 μl reaction volume). Depending on the target species, this was achieved at the highest or the second highest dilution step, where one or five target copies per μl extract were expected, respectively. At these low concentrations, the stochastic nature of PCR causes some variation in detection success [19] and based on the number of replicates and the orders of magnitude covered in the dilution-series experiment, it was not possible to further refine the LOD and LOQ for each target species [24,25]. Nevertheless, celPCR showed sufficient sensitivity to detect target DNA in field-collected samples and copy numbers in field-collected samples could be estimated with the model obtained from the dilution series experiment, even though some signals were below the lower limit of the dilution series. Our results were consistent between dPCR and multiplex celPCR, except for two field-collected samples, which tested negative in multiplex celPCR, but contained < 0.25 copies per μl extract in dPCR. Such low-concentration positives (below the LOD) have been previously observed in dPCR [24] and should be re-tested for further evaluation as these can be true positives, but also result from background signals of fluorescing foreign particles [61,62]. If target DNA is expected to be present mostly at very low concentrations (e.g. < 10 copies/μl), it is, however, possible to pre-amplify target DNA with a preceding PCR using general primers, when the aim is a presence/absence evaluation [41].

For all PCR platforms and visualization methods in this study, a threshold is used to differentiate negative from positive results. In dPCR, this separates positive from negative droplets [10,46], whereas the lowest fluorescence signal distinctly different from background noise needs to be specified for both qPCR [24] and capillary electrophoresis [17]. The use of EvaGreen Supermix made results directly comparable between celPCR and dPCR since the same primer pairs were used. However, this dPCR chemistry should be used with care, as the levels of background fluorescence can vary between field-collected samples.

The possibility for quantification via multiplex celPCR is appealing for target eDNA detection from high sample numbers as reactions can usually be set up for simultaneous detection of five to ten species [17,29,34]. Especially commercial providers of eDNA services and smaller laboratories, which do not always have access to the newest technological advances, could benefit from this sensitive and cost-efficient approach (Table 1) when handling large sample numbers. The semi-quantitative assessment of eDNA levels contained in field-collected samples is possible via celPCR after designing specific primers, optimizing the celPCR for maximum sensitivity, and evaluating performance in a subset of field-collected samples. Nevertheless, direct inference of the DNA concentration in the sample and absolute quantitative comparisons between target species are only possible when accounting for primer effects, inhibition, and calibrating celPCR results using dPCR or other methods of quantifying target DNA. Despite this limitation, multiplex celPCR is a highly sensitive and broadly applicable tool for the detection and quantification of eDNA and will enable efficient large-scale screenings in the context of species distribution monitoring at more affordable costs.

## Supporting information

S1 File Alignment of target species, non-target species, and primers. (FAS)

S2 File Linear models with Relative Fluorescence Units (RFU) from singleplex (SP) celPCR as predictor for RFU from multiplex celPCR. (PDF)

S3 File Linear models with Relative Fluorescence Units (RFU) as predictor for *ln*-transformed copy numbers per μl extract. (PDF)

S4 File The best performing linear mixed-effects model (LMMmax70) derived from the 200 70% subsets, which were tested on the respective 30% subsets for their accuracy. (PDF)

S5 File The field-sample-based linear models per primer pair and respective target species. (PDF)

S6 File Per primer pair and respective target species, the linear models describing the relationship between observed and predicted copies per μl extract based on the entire dilution series experiment and LMM_full_. (PDF)

S7 File (PDF)
