# Supplementary material for: Endpoint PCR coupled with capillary electrophoresis (celPCR) provides sensitive and quantitative measures of environmental DNA in singleplex and multiplex reactions
Source: PLoS One. 2021 Jul 23;16(7):e0254356. doi: 10.1371/journal.pone.0254356 (PMC8301609; doi:10.1371/journal.pone.0254356)
Supplement: S2 File — (PDF) [file pone.0254356.s002.pdf]

Endpoint PCR coupled with capillary electrophoresis (celPCR) provides sensitive and quantitative measures of environmental DNA in singleplex and multiplex reactions

Supporting Information 2

Bettina Thalinger[1,2,3*], Yannick Pütz[1] & Michael Traugott[1,4]

[1] Department of Zoology, University of Innsbruck, Technikerstr. 25, 6020, Innsbruck, Austria

[2] Centre for Biodiversity Genomics, University of Guelph, 50 Stone Road E, N1G 2W1, Guelph, Ontario, Canada

[3] Department of Integrative Biology, College of Biological Science, University of Guelph, 50 Stone Road E, N1G 2W1, Guelph, Ontario, Canada.

[4] Sinsoma GmbH, Lannes 6, 6176 Voels, Austria

***Corresponding author:**

Bettina Thalinger, bettina.thalinger@gmail.com

Centre for Biodiversity Genomics, University of Guelph, 50 Stone Road E, N1G 2W1, Guelph, Ontario, Canada

S2 Table: Linear models with Relative Fluorescence Units (RFU) from singleplex (SP) celPCR as predictor for RFU from multiplex celPCR. Per species, models are based on the mean values per dilution step. Columns describe the target species, adjusted R^2, the predictor variable, parameter estimates, standard errors, 95%-CIs, t-value, and p-value.

**species**	**R^2 adj.**	**predictor variable**	**parameter estimate**	**SE**	**lower 95% CI**	**upper 95% CI**	**t-value**	**p-value**
C. gobio	0.68	intercept	0.59	0.41	-0.27	1.44	1.44	0.17
		SP RFU	0.90	0.14	0.61	1.19	6.44	<0.001***
O. mykiss	0.93	intercept	0.02	0.09	-0.17	0.21	0.26	0.797
		SP RFU	0.98	0.06	0.85	1.10	16.08	<0.001***
S. cephalus	0.79	intercept	0.79	0.18	0.41	1.16	4.41	<0.001***
		SP RFU	0.51	0.06	0.38	0.63	8.63	<0.001***
S. fontinalis	0.91	intercept	0.12	0.10	-0.09	0.33	1.19	0.25
		SP RFU	0.83	0.06	0.71	0.96	13.79	<0.001***
S. trutta	0.92	intercept	0.01	0.11	-0.22	0.25	0.13	0.898
		SP RFU	1.01	0.07	0.86	1.16	14.04	<0.001***
T. thymallus	0.73	intercept	0.62	0.30	-0.001	1.24	2.10	0.05
		SP RFU	0.65	0.09	0.46	0.84	7.23	<0.001***
