# Supplementary material for: Endpoint PCR coupled with capillary electrophoresis (celPCR) provides sensitive and quantitative measures of environmental DNA in singleplex and multiplex reactions
Source: PLoS One. 2021 Jul 23;16(7):e0254356. doi: 10.1371/journal.pone.0254356 (PMC8301609; doi:10.1371/journal.pone.0254356)
Supplement: S3 File — (PDF) [file pone.0254356.s003.pdf]

Endpoint PCR coupled with capillary electrophoresis (celPCR) provides sensitive and quantitative measures of environmental DNA in singleplex and multiplex reactions

Supporting Information 3

Bettina Thalinger[1,2,3*], Yannick Pütz[1] & Michael Traugott[1,4]

[1] Department of Zoology, University of Innsbruck, Technikerstr. 25, 6020, Innsbruck, Austria

[2] Centre for Biodiversity Genomics, University of Guelph, 50 Stone Road E, N1G 2W1, Guelph, Ontario, Canada

[3] Department of Integrative Biology, College of Biological Science, University of Guelph, 50 Stone Road E, N1G 2W1, Guelph, Ontario, Canada.

[4] Sinsoma GmbH, Lannes 6, 6176 Voels, Austria

***Corresponding author:**

Bettina Thalinger, bettina.thalinger@gmail.com

Centre for Biodiversity Genomics, University of Guelph, 50 Stone Road E, N1G 2W1, Guelph, Ontario, Canada

S3 Table: Linear models with Relative Fluorescence Units (RFU) as predictor for *ln*-transformed copy numbers per µl extract. Models for both singleplex and multiplex celPCR data were calculated using the mean values per dilution step and without including target species identity as categorical variable. Columns describe the source of the predicted values, the target species, adjusted R^2, the predictor variable, its parameter estimates, standard errors, 95%-CIs, t-value, and p-value.

**species**	**R^2 adj.**	**predictor variable**	**parameter estimate**	**SE**	**lower 95% CI**	**upper 95% CI**	**t-value**	**p-value**
Singleplex PCR	0.55	intercept	3.14	0.27	2.61	3.68	11.52	<0.001***
		RFU	1.37	0.11	1.15	1.59	12.24	<0.001***
Multiplex PCR	0.51	intercept	3.10	0.30	2.51	3.70	10.31	<0.001***
		RFU	1.49	0.13	1.22	1.75	11.11	<0.001***
