# Supplementary material for: Endpoint PCR coupled with capillary electrophoresis (celPCR) provides sensitive and quantitative measures of environmental DNA in singleplex and multiplex reactions
Source: PLoS One. 2021 Jul 23;16(7):e0254356. doi: 10.1371/journal.pone.0254356 (PMC8301609; doi:10.1371/journal.pone.0254356)
Supplement: S4 File — (PDF) [file pone.0254356.s004.pdf]

Endpoint PCR coupled with capillary electrophoresis (celPCR) provides sensitive and quantitative measures of environmental DNA in singleplex and multiplex reactions

Supporting Information 4

Bettina Thalinger[1,2,3*], Yannick Pütz[1] & Michael Traugott[1,4]

[1] Department of Zoology, University of Innsbruck, Technikerstr. 25, 6020, Innsbruck, Austria

[2] Centre for Biodiversity Genomics, University of Guelph, 50 Stone Road E, N1G 2W1, Guelph, Ontario, Canada

[3] Department of Integrative Biology, College of Biological Science, University of Guelph, 50 Stone Road E, N1G 2W1, Guelph, Ontario, Canada.

[4] Sinsoma GmbH, Lannes 6, 6176 Voels, Austria

***Corresponding author:**

Bettina Thalinger, bettina.thalinger@gmail.com

Centre for Biodiversity Genomics, University of Guelph, 50 Stone Road E, N1G 2W1, Guelph, Ontario, Canada

S4 Table: The best performing linear mixed-effects model (LMM_{max70}) derived from the 200 70% subsets, which were tested on the respective 30% subsets for their accuracy.

LMM_{max70}	Random effects			Variance		Standard deviation	
	intercept			0.69		0.83	
	Mean MP PCR RFU			0.80		0.90	
	**Fixed effects**	**parameter estimate**	**lower 95% CI**	**upper 95% CI**	**t-value**	**p-value**	
	intercept	0.99	0.19	1.80	2.46	< 0.05	*
	Mean MP PCR RFU	2.75	1.99	3.51	7.21	< 0.001	***
	**Estimated deviation**	**species**	**intercept**	**Mean MP PCR RFU**			
		C. gobio	0.48	–1.39			
		O. mykiss	0.71	0.25			
		S. cephalus	–1.21	0.14			
		S. fontinalis	–0.08	1.00			
		S. trutta	0.36	0.60			
		T. thymallus	–0.26	–059			
