# Supplementary material for: Endpoint PCR coupled with capillary electrophoresis (celPCR) provides sensitive and quantitative measures of environmental DNA in singleplex and multiplex reactions
Source: PLoS One. 2021 Jul 23;16(7):e0254356. doi: 10.1371/journal.pone.0254356 (PMC8301609; doi:10.1371/journal.pone.0254356)
Supplement: S5 File — (PDF) [file pone.0254356.s005.pdf]

Endpoint PCR coupled with capillary electrophoresis (celPCR) provides sensitive and quantitative measures of environmental DNA in singleplex and multiplex reactions

Supporting Information 5

Bettina Thalinger[1,2,3*], Yannick Pütz[1] & Michael Traugott[1,4]

[1] Department of Zoology, University of Innsbruck, Technikerstr. 25, 6020, Innsbruck, Austria

[2] Centre for Biodiversity Genomics, University of Guelph, 50 Stone Road E, N1G 2W1, Guelph, Ontario, Canada

[3] Department of Integrative Biology, College of Biological Science, University of Guelph, 50 Stone Road E, N1G 2W1, Guelph, Ontario, Canada.

[4] Sinsoma GmbH, Lannes 6, 6176 Voels, Austria

***Corresponding author:**

Bettina Thalinger, bettina.thalinger@gmail.com

Centre for Biodiversity Genomics, University of Guelph, 50 Stone Road E, N1G 2W1, Guelph, Ontario, Canada

S5 Table. Per primer pair and respective target species, the field-sample-based linear models are listed. They describe the relationship between multiplex-based Relative Fluorescence Units (MP RFU) and *ln*-transformed copies per µl extract. Columns describe the target species, adjusted R^2, the predictor variable, its parameter estimates, standard errors, 95%-CIs, t-value, and p-value.

species	R^2 adj.	predictor variable	parameter estimate	SE	lower 95% CI	upper 95% CI	t-value	p-value
C. gobio	0.82	intercept	0.69	0.25	0.17	1.21	2.76	0.01*
		MP RFU	2.06	0.22	1.61	2.52	9.47	<0.001***
O. mykiss	0.74	intercept	0.60	0.36	-0.14	1.34	1.69	0.11
		MP RFU	3.56	0.43	2.67	4.45	8.26	<0.001***
S. cephalus	0.82	intercept	0.09	0.35	-0.65	0.84	0.26	0.80
		MP RFU	4.33	0.46	3.38	5.28	9.51	<0.001***
S. fontinalis	0.63	intercept	-0.25	0.53	-1.34	0.84	-0.47	0.65
		MP RFU	5.35	0.84	3.61	7.08	6.39	<0.001***
S. trutta	0.37	intercept	2.00	0.53	0.90	3.10	3.75	0.001**
		MP RFU	2.26	0.60	1.02	3.49	3.80	<0.001***
T. thymallus	0.13	intercept	1.09	0.72	-0.40	2.57	1.52	0.14
		MP RFU	2.39	1.15	-0.004	4.78	2.07	0.05
