# Supplementary material for: Endpoint PCR coupled with capillary electrophoresis (celPCR) provides sensitive and quantitative measures of environmental DNA in singleplex and multiplex reactions
Source: PLoS One. 2021 Jul 23;16(7):e0254356. doi: 10.1371/journal.pone.0254356 (PMC8301609; doi:10.1371/journal.pone.0254356)
Supplement: S6 File — (PDF) [file pone.0254356.s006.pdf]

Endpoint PCR coupled with capillary electrophoresis (celPCR) provides sensitive and quantitative measures of environmental DNA in singleplex and multiplex reactions

Supporting Information 6

Bettina Thalinger[1,2,3*], Yannick Pütz[1] & Michael Traugott[1,4]

[1] Department of Zoology, University of Innsbruck, Technikerstr. 25, 6020, Innsbruck, Austria

[2] Centre for Biodiversity Genomics, University of Guelph, 50 Stone Road E, N1G 2W1, Guelph, Ontario, Canada

[3] Department of Integrative Biology, College of Biological Science, University of Guelph, 50 Stone Road E, N1G 2W1, Guelph, Ontario, Canada.

[4] Sinsoma GmbH, Lannes 6, 6176 Voels, Austria

***Corresponding author:**

Bettina Thalinger, bettina.thalinger@gmail.com

Centre for Biodiversity Genomics, University of Guelph, 50 Stone Road E, N1G 2W1, Guelph, Ontario, Canada

S6 Table. Per primer pair and respective target species, the linear models describing the relationship between observed and predicted copies per µl extract based on the entire dilution series experiment and LMM_{full} (Table 2). Models were calculated separately for each species using predictions from multiplex-based Relative Fluorescence Units (MP RFU). Columns describe the target species, adjusted R^2, the predictor variable (i.e the observed *ln*-transformed copy numbers), their parameter estimates, standard errors, 95%-CIs, t-value, and p-value.

species	R^2 adj.	predictor variable	parameter estimate	SE	lower 95% CI	upper 95% CI	t-value	p-value
C. gobio	0.79	intercept	0.96	0.60	-0.30	2.23	1.60	0.13
		ln(copies / µl)	0.83	0.10	0.62	1.03	8.42	<0.001***
O. mykiss	0.96	intercept	-0.08	0.27	-0.66	0.49	-0.30	0.77
		ln(copies / µl)	1.01	0.04	0.91	1.10	22.60	<0.001***
S. fontinalis	0.95	intercept	0.63	0.31	-0.02	1.27	2.04	0.06
		ln(copies / µl)	0.90	0.04	0.80	1.00	18.68	<0.001***
S. trutta	0.95	intercept	0.16	0.32	-0.51	0.83	0.51	0.62
		ln(copies / µl)	0.97	0.05	0.87	1.07	19.85	<0.001***
S. cephalus	0.80	intercept	1.92	0.54	0.79	3.04	3.57	<0.01**
		ln(copies / µl)	0.71	0.08	0.54	0.88	8.72	<0.001***
T. thymallus	0.90	intercept	0.67	0.46	-0.29	1.63	1.45	0.16
		ln(copies / µl)	0.90	0.07	0.76	1.04	13.30	<0.001***
