# Supplementary material for: Endpoint PCR coupled with capillary electrophoresis (celPCR) provides sensitive and quantitative measures of environmental DNA in singleplex and multiplex reactions
Source: PLoS One. 2021 Jul 23;16(7):e0254356. doi: 10.1371/journal.pone.0254356 (PMC8301609; doi:10.1371/journal.pone.0254356)
Supplement: S7 File — (PDF) [file pone.0254356.s007.pdf]

Endpoint PCR coupled with capillary electrophoresis (celPCR) provides sensitive and quantitative measures of environmental DNA in singleplex and multiplex reactions

Supporting Information 2

Bettina Thalinger[1,2,3], Yannick Pütz[1] & Michael Traugott[1,4]

[1] Department of Zoology, University of Innsbruck, Technikerstr. 25, 6020, Innsbruck, Austria

[2] Centre for Biodiversity Genomics, University of Guelph, 50 Stone Road E, N1G 2W1, Guelph, Ontario, Canada

[3] Department of Integrative Biology, College of Biological Science, University of Guelph, 50 Stone Rd E (N1G 2W1), Guelph, Ontario, Canada.

[4] Sinsoma GmbH, Lannes 6, 6176 Voels, Austria

***Corresponding author:**

Bettina Thalinger, bettina.thalinger@gmail.com

Centre for Biodiversity Genomics, University of Guelph, 50 Stone Road E, N1G 2W1, Guelph, Ontario, Canada

SI2 Table I: Linear models with Relative Fluorescence Units (RFU) from singleplex (SP) celPCR as predictor for RFU from multiplex celPCR. Per species, models are based on the mean values per dilution step. Columns describe the target species, adjusted R², the predictor variable, parameter estimates, standard errors, 95%-CIs, t-value, and p-value.

**species**	**R² adj.**	**predictor variable**	**parameter estimate**	**SE**	**lower 95% CI**	**upper 95% CI**	**t-value**	**p-value**
C. gobio	0.68	intercept	0.59	0.41	-0.27	1.44	1.44	0.17
		SP RFU	0.90	0.14	0.61	1.19	6.44	<0.001***
O. mykiss	0.93	intercept	0.02	0.09	-0.17	0.21	0.26	0.797
		SP RFU	0.98	0.06	0.85	1.10	16.08	<0.001***
S. cephalus	0.79	intercept	0.79	0.18	0.41	1.16	4.41	<0.001***
		SP RFU	0.51	0.06	0.38	0.63	8.63	<0.001***
S. fontinalis	0.91	intercept	0.12	0.10	-0.09	0.33	1.19	0.25
		SP RFU	0.83	0.06	0.71	0.96	13.79	<0.001***
S. trutta	0.92	intercept	0.01	0.11	-0.22	0.25	0.13	0.898
		SP RFU	1.01	0.07	0.86	1.16	14.04	<0.001***
T. thymallus	0.73	intercept	0.62	0.30	-0.001	1.24	2.10	0.05
		SP RFU	0.65	0.09	0.46	0.84	7.23	<0.001***

SI2 Table II: Linear models with Relative Fluorescence Units (RFU) as predictor for *ln*-transformed copy numbers per µl extract. Models for both singleplex and multiplex celPCR data were calculated using the mean values per dilution step and without including target species identity as categorical variable. Columns describe the source of the predicted values, the target species, adjusted R^2, the predictor variable, its parameter estimates, standard errors, 95%-CIs, t-value, and p-value.

species	R^2 adj.	predictor variable	parameter estimate	SE	lower 95% CI	upper 95% CI	t-value	p-value
Singleplex PCR	0.55	intercept	3.14	0.27	2.61	3.68	11.52	<0.001***
		RFU	1.37	0.11	1.15	1.59	12.24	<0.001***
Multiplex PCR	0.51	intercept	3.10	0.30	2.51	3.70	10.31	<0.001***
		RFU	1.49	0.13	1.22	1.75	11.11	<0.001***

SI2 Table III: The best performing linear mixed-effects model (LMM_{max70}) derived from the 200 70% subsets, which were tested on the respective 30% subsets for their accuracy.

LMM_{max70}	Random effects			Variance		Standard deviation	
	intercept			0.69		0.83	
	Mean MP PCR RFU			0.80		0.90	
	**Fixed effects**	**parameter estimate**	**lower 95% CI**	**upper 95% CI**	**t-value**	**p-value**	
	intercept	0.99	0.19	1.80	2.46	< 0.05	*
	Mean MP PCR RFU	2.75	1.99	3.51	7.21	< 0.001	***
	**Estimated deviation**	**species**	**intercept**	**Mean MP PCR RFU**			
		C. gobio	0.48	–1.39			
		O. mykiss	0.71	0.25			
		S. cephalus	–1.21	0.14			
		S. fontinalis	–0.08	1.00			
		S. trutta	0.36	0.60			
		T. thymallus	–0.26	–059			

SI2 Table IV: Per primer pair and respective target species, the field-sample-based linear models are listed. They describe the relationship between multiplex-based Relative Fluorescence Units (MP RFU) and *ln*-transformed copies per µl extract. Columns describe the target species, adjusted R^2, the predictor variable, its parameter estimates, standard errors, 95%-CIs, t-value, and p-value.

**species**	**R^2 adj.**	**predictor variable**	**parameter estimate**	**SE**	**lower 95% CI**	**upper 95% CI**	**t-value**	**p-value**
C. gobio	0.82	intercept	0.69	0.25	0.17	1.21	2.76	0.01*
		MP RFU	2.06	0.22	1.61	2.52	9.47	<0.001***
O. mykiss	0.74	intercept	0.60	0.36	-0.14	1.34	1.69	0.11
		MP RFU	3.56	0.43	2.67	4.45	8.26	<0.001***
S. cephalus	0.82	intercept	0.09	0.35	-0.65	0.84	0.26	0.80
		MP RFU	4.33	0.46	3.38	5.28	9.51	<0.001***
S. fontinalis	0.63	intercept	-0.25	0.53	-1.34	0.84	-0.47	0.65
		MP RFU	5.35	0.84	3.61	7.08	6.39	<0.001***
S. trutta	0.37	intercept	2.00	0.53	0.90	3.10	3.75	0.001**
		MP RFU	2.26	0.60	1.02	3.49	3.80	<0.001***
T. thymallus	0.13	intercept	1.09	0.72	-0.40	2.57	1.52	0.14
		MP RFU	2.39	1.15	-0.004	4.78	2.07	0.05

SI2 Table V: Per primer pair and respective target species, the linear models describing the relationship between observed and predicted copies per µl extract based on the entire dilution series experiment and LMM_{full} (Table 2). Models were calculated separately for each species using predictions from multiplex-based Relative Fluorescence Units (MP RFU). Columns describe the target species, adjusted R^2, the predictor variable (i.e the observed *ln*-transformed copy numbers), their parameter estimates, standard errors, 95%-CIs, t-value, and p-value.

species	R^2 adj.	predictor variable	parameter estimate	SE	lower 95% CI	upper 95% CI	t-value	p-value
C. gobio	0.79	intercept	0.96	0.60	-0.30	2.23	1.60	0.13
		ln(copies / µl)	0.83	0.10	0.62	1.03	8.42	<0.001***
O. mykiss	0.96	intercept	-0.08	0.27	-0.66	0.49	-0.30	0.77
		ln(copies / µl)	1.01	0.04	0.91	1.10	22.60	<0.001***
S. fontinalis	0.95	intercept	0.63	0.31	-0.02	1.27	2.04	0.06
		ln(copies / µl)	0.90	0.04	0.80	1.00	18.68	<0.001***
S. trutta	0.95	intercept	0.16	0.32	-0.51	0.83	0.51	0.62
		ln(copies / µl)	0.97	0.05	0.87	1.07	19.85	<0.001***
S. cephalus	0.80	intercept	1.92	0.54	0.79	3.04	3.57	<0.01**
		ln(copies / µl)	0.71	0.08	0.54	0.88	8.72	<0.001***
T. thymallus	0.90	intercept	0.67	0.46	-0.29	1.63	1.45	0.16
		ln(copies / µl)	0.90	0.07	0.76	1.04	13.30	<0.001***
